# THE BURDEN OF ENGAGING IN VOLUNTEER ATTENUATE THE RELATIONSHIP BETWEEN VOLUNTEER ENGAGEMENT AND HEALTH OUTCOMES

**DOI:** 10.1093/geroni/igz038.524

**Published:** 2019-11-08

**Authors:** Yuta Nemoto, Ryota Sakurai, Masami Hasebe, Kumiko Nonaka, Hiroko Mtsunaga, Yoshinori Fujiwara

**Affiliations:** 1 Tokyo Metropolitan Institute of Gerontology, Itabashi, Tokyo, Japan; 2 Tokyo Metropolitan Institute of Gerontology, Tokyo, Japan

## Abstract

This study examined the interaction of participating in volunteer activity and its burden on health outcomes. A community-based cross-sectional study was conducted in 2018. Of 8426 older adults aged 65 and over, 5232 individuals were included in the analyses (response rate: 62.1%). Health outcomes included self-rated health (SRH), mental health (The World Health Organization Five Well-Being Index: WHO-5), and Instrumental Activities of Daily Living (Tokyo Metropolitan Institute of Gerontology Index of Competence: TMIG-IC) as dependent variables. Independent variables included engagement in volunteer activity and its burden. These variables were combined and classified into three groups: non-participants; participants with burden; and participants without burden. Covariates included age, gender, educational attainments, economic status, and living arrangement. Logistic regression analysis and analysis of covariates were conducted to examine the interaction of volunteer activity and its burden on health outcomes. Of 5232 older adults, 76.3% of subjects were non-participants, 3.4% were participants with burden, and 20.3% were participants without burden. Multivariate analysis showed that non-participants were more likely to have poor health outcomes compared with participants with burden. Moreover, participants without burden were more likely to have better health outcomes (SRH: Odds Ratio [OR] = 1.92, 95% Confidence Interval [CI] = 1.70 to 2.17, WHO-5: OR = 1.69, 95% CI = 1.51 to 1.88, TMIG-IC: Coefficient = 0.36, 95% CI = 0.10 to 0.62). Our findings suggest that volunteer activity is related to better health regardless of their burden. However, burden of volunteer engagement might attenuate the relationships between volunteer activity and health outcomes.

